# The field of psychiatry is in crisis. The case for causal modelling in observational data as a supplement to psychiatric epidemiology and clinical trials

**DOI:** 10.1192/j.eurpsy.2022.1523

**Published:** 2022-09-01

**Authors:** A. Mykletun

**Affiliations:** Haukeland University Hospital, Mental Health Services, Bergen, Norway

**Keywords:** Epidemiology, systematic review

## Abstract

**Introduction:**

The field of psychiatry is in a crisis. Developments in pharmacology and psychotherapy, reforms in services, increased spending and reduced treatment-gap have not substantially improved prognosis for patients in psychiatry. Mental disorder remains lethal short-term and disabling long term. In comparison, prognosis has improved dramatically in oncology and cardiology. Controversies in psychiatry are causing variation in clinical practice between hospitals, even within single-provider health systems. There is, for example, variation in rates of ADHD, use of coercive measures, and medication (type of drugs, dose and duration of medication). Current empirical methods are incapable of solving the major controversies in psychiatry. Epidemiology struggles with residual confounding, bias and reverse causality. Randomized controlled trials are expensive and time-consuming. Ethics may also be a barrier for clinical studies investigating variation in clinical practice. From a health management point of view, variation in clinical services within a single-provider system is usually indicative of variation in quality. However, the variability in service delivery caused by these controversies creates a lottery-like situation for the individual patient, who is generally unaware of the crisis in psychiatry, and blinded to the ongoing lottery.

**Objectives:**

We will present a third empirical approach beyond randomized controlled trials and epidemiology which may help solve the crisis.

**Methods:**

A systematic review of preference-based instrument variable analyses.

**Results:**

We identified relevant high quality 185 studies, though almost none in mental health.

**Conclusions:**

Causal modelling in observational data has potential as a third paradigm beyond RCTs and epidemiology, and may help solve the crisis in psychiatry.

**Disclosure:**

No significant relationships.

